# Evaluating OpenFace: an open-source automatic facial comparison algorithm for forensics

**DOI:** 10.1080/20961790.2018.1523703

**Published:** 2018-10-09

**Authors:** Angeliki Fydanaki, Zeno Geradts

**Affiliations:** Netherlands Forensic Institute, University of Amsterdam, Amsterdam, The Netherlands

**Keywords:** Forensic sciences, digital forensic, face comparison, OpenFace, deep learning

## Abstract

This article studies the application of models of OpenFace (an open-source deep learning algorithm) to forensics by using multiple datasets. The discussion focuses on the ability of the software to identify similarities and differences between faces based on images from forensics. Experiments using OpenFace on the Labeled Faces in the Wild (LFW)-raw dataset, the LFW-deep funnelled dataset, the Surveillance Cameras Face Database (SCface) and ForenFace datasets showed that as the resolution of the input images worsened, the effectiveness of the models degraded. In general, the effect of the quality of the query images on the efficiency of OpenFace was apparent. Therefore, OpenFace in its current form is inadequate for application to forensics, but can be improved to offer promising uses in the field.

## Introduction

Over the past decade, algorithms for automatic facial analysis have garnered the interest of the biometrics community. However, none of the ones proposed thus far has been able to generate results that compare in accuracy to manual identification of faces by a human. OpenFace is an open-source toolkit based on the FaceNet algorithm for automatic facial identification that was created by Google [1]. It has been developed and shared as an open-source software by Brandon Amos in Satya’s research group at Carnegie Mellon University [2]. The main advantages of OpenFace are that it does not require many human resources, it has yielded impressive performance on the Labeled Faces in the Wild (LFW) benchmark, and it is open source.

OpenFace has multiple potential applications to forensics, such as facial recognition of suspects of crimes and people reported missing. Moreover, it can be used to identify dead bodies.

The purpose of this study is to examine whether OpenFace is sufficiently efficient enough for use in forensics by assessing its performance.

## Materials and methods

There are three main tasks related to digital facial identification: (1) verification, (2) recognition and (3) clustering of faces. FaceNet and OpenFace can perform these tasks based on stable images and real-time Web videos [3,4]. FaceNet is based on Euclidean embedding per image, which uses the deep neural net (DNN) for the representation/embedding of a query face on a 128-dimensional (128D) unit hypersphere [1]. Furthermore, squared L2 distances are used to determine similarities among pairs of faces posed as queries. Moreover, recognition is performed by using *k*-nearest neighbours (*k*-NN) classification algorithm, whereas the clustering of the similar faces is effected using the well-known *k*-means clustering or agglomerative clustering. Moreover, a triplet-based loss function used during training, based on the large-margin nearest-neighbour (LMNN) classification [5], ensures that the output of FaceNet is correctly used. The output is composed of matching and non-matching faces [5,6]. In general, it minimizes the relevant distance when the same face appears in both query images and maximizes it when different the faces appearing in the pair of query images are different. This is based on Equation (1), where $\chi_{i}^{\alpha },\chi_{i}^{p}\wedge \chi_{i}^{n}$ are the anchors, positive images and negative images, respectively; *T* represents all possible sets of triplets in the training set with *N* entities; and α is the margin between the positive and negative pairs of faces:

(1) $$\begin{array}{l} {\left\|x_{i}^{a}-x_{i}^{p}\right\|}_{2}^{2}+\alpha {\left\|x_{i}^{a}-x_{i}^{n}\right\|}_{2}^{2},\\ \forall \left(x_{i}^{a},x_{i}^{p},x_{i}^{n}\right)\in T. \end{array}$$

The minimized loss is described in Equation (2):

(2) $${\sum_{i}^{N}\left[{\left\|f(x_{i}^{a})-f(x_{i}^{p})\right\|}_{2}^{2}-{\left\|f(x_{i}^{a})-f(x_{i}^{n})\right\|}_{2}^{2}+\alpha \right]}_{+}.$$

(3) $$\text{argma}x_{x_{i}^{p}}{\left\|f(x_{i}^{a})-f(x_{i}^{p})\right\|}_{2}^{2}.$$

(4) $$\text{argmin}{}_{x_{i}^{n}}{\left\|f(x_{i}^{a})-f(x_{i}^{n})\right\|}_{2}^{2}.$$

(5) $${\left\|f(x_{i}^{a})-f(x_{i}^{p})\right\|}_{2}^{2}<{\left\|f(x_{i}^{a})-f(x_{i}^{n})\right\|}_{2}^{2}.$$

A critical step in this triplet loss function is the selection of triplets that can help improve method. To do so, we select hard positives and negatives according to Equations (3) and (4), respectively. The convolutional neural network (CNN) training of the models, as described in [1], is carried out based on two methods, stochastic gradient descent (SGD) with standard backpropagation [7,8] and AdaGrad [9]. In general, there are two types of models, the differences between which lie in their floating-point operations per second (FLOPS) and the parameters used [10,11].

**Figure 1.**
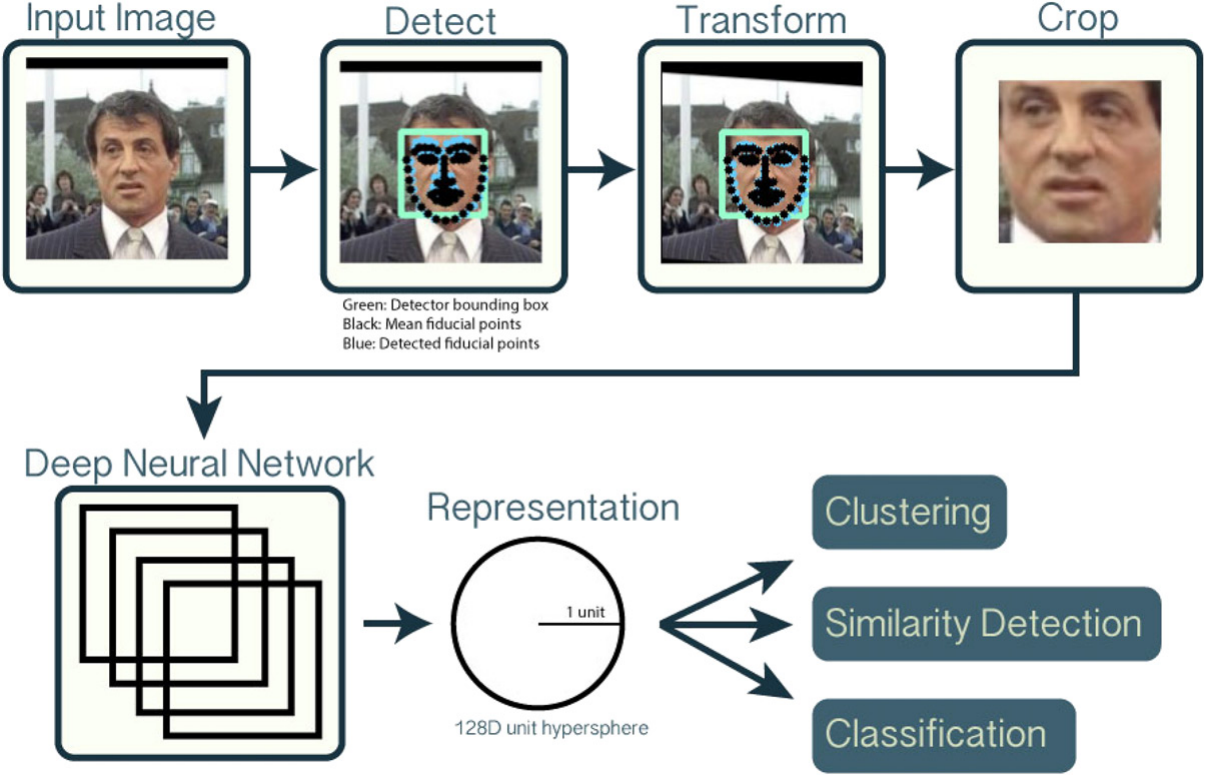
An example of the OpenFace procedure ADDIN.

An example of the whole OpenFace procedure is given in Figure 1 using an image of the actor Sylvester Stallone from the LFW datasets.

The authors in [1] claimed that a threshold of 1,1 is sufficient for the correct classification of query faces, whereas 0,0 corresponds to identical faces and 4,0 to completely opposite faces [1].

The necessary changes to the OpenFace code were made so that it could be used with the chosen databases, instead of to using a comparison of the images one by one. The main change in the code in this aspect was the omission of the itertools called combinations_with_replacement (iteration, *r*) in the initial code [12]. This function returns combinations of the inputs of a specific length (input), where the replacement of elements is allowed. For instance, if the input “ABC” for the iteration *r* is two, the following combinations are returned: AA, AB, AC, BB, BC and CC.

This means that the number of the returned elements is

(6) (n+r−1)!/r!/(n−1)!

where *n* is the input dataset, where *n* > 0, and *r* is the length of the combinations. For the purpose of this research, the best means of comparison is to modify the implementation of the relevant file such that the first image is compared against the rest. Moreover, additional modifications to the initial code involve saving image names, the values of the relevant distances in an array, and sorting them in ascending order for easier examination of the most relevant faces in the images. It should be mentioned that when the model cannot find a face in an image, the relevant distance is initialized to 4.0, which is the maximum possible distance. Finally, the modified program can accept more than one input image to compare against the rest of the dataset. Once the classification stops when the detector cannot find a face on an image, we manually remove all these images and run the algorithm again on the remainder of the image dataset.

## Experiments

We verified the performance of the method above on an NVIDIA Corporation GM204 [GeForce GTX 980] graphics processing unit (GPU) with Ubuntu 14.04. Moreover, various open-source materials are needed to properly use OpenFace, namely OpenCV [13], Torch [14], Git [15], Docker [16] and Dlib [17].

### Datasets

The input data were one of the most important aspects of this study because the images used in forensic applications, such as from surveillance cameras, are low resolution, or are left with poor resolution following the detection and localization of the faces in them.

For this study, multiple datasets were used:

- LFW benchmark [18], with two types of data: raw and deep-funnelled images;
- Surveillance Cameras Face Database (SCface) [19], with case scenarios from typical forensic cases with frontal and profile images from surveillance cameras; and
- ForenFace [20], with case scenarios from typical forensics’ cases with frontal, side, and profile facial images captured using surveillance cameras, and underwent multiple modifications, such as being cropped and turned.

### Verification on LFW

The first part of the experiments involved the verification of the reported results of OpenFace using the LFW benchmark [4]. Only four pre-trained open-source NN4 models were available. Table 1 lists the parameters and landmark indices for each model [4]. They were trained on two large datasets: FaceScrub [21], which contained over 100 000 images of 530 people; and CASIA-WebFace [22], which consisted of 500 000 images of 10 000 people.

**Table 1.** Parameters of OpenFace and landmark indices per model [4].

Model	Number of parameters	Alignment
nn4.v1	6 959 088	INNER EYES AND BOTTOM LIP
nn4.v2	6 959 088	OUTER EYES AND NOSE
nn4.small1.v1	5 579 520	OUTER EYES AND NOSE
nn4.small2.v1	3 733 968	OUTER EYES AND NOSE

Our experiments were conducted on the Docker machine on an eight-core, 3.70 GHz central processing unit (CPU) and a Tesla K40 GPU using OpenBLAS [24]. The performance of the models was calculated by averaging over 500 forward passes with util/profile-network.lua, and the results are shown in Table 2 [4].

**Table 2.** Runtime of OpenFace on both central processing unit (CPU) and graphics processing unit (GPU) per model [25].

Model	Runtime ($\bar{x}$ ± s, ms)	
	CPU	GPU
nn4.v1	75.67 ± 19.97	21.00 ± 6.71
nn4.v2	82.74 ± 19.96	20.82 ± 6.03
nn4.small1.v1	69.58 ± 16.17	15.90 ± 5.18
nn4.small2.v1	58.90 ± 15.36	13.72 ± 4.64

When the models used the deep-funnelled LFW images, they could not detect a face or landmark using a dlib of 58 for 13 233 images. Our experiments on the NVIDIA GM204 [GeForce GTX 980] GPU with Ubuntu 14.04 for the available evaluation (lfw.py) yielded results (Table 3) that show the accuracy and the area under curve (AUC) of each model on the LFW dataset in comparison with FaceNet and the AUC reported in [1]. An important aspect of this evaluation was that eight processes were used by the researchers in [1], which was the number of random processes used for our verification of the results reported by Brandon Amos concerning Satya's research group at Carnegie Mellon University [2,4].

**Table 3.** The accuracy and area under the curve (AUC) of each model on OpenFace, and accuracy and AUC of FaceNet as reported in [1].

Model	Accuracy ($\bar{x}$ ± s)	AUC
nn4.v1	0.761 2 ± 0.018 9	0.853
nn4.v2	0.915 7 ± 0.015 2	0.966
nn4.small2.v1	0.929 2 ± 0.013 4	0.973
nn4.small1.v1	0.921 0 ± 0.016 0	0.973

## Results

The model was evaluated based on the equal error rate (EER), receiver operating characteristic (ROC) and the AUC [25–27]. To create a new evaluation code, an important requirement is that when the model is unable to find a face in the input image, it should assign the greatest possible distance to it (4.00) because more images can be used in this way for the assessment of the models in each dataset, which renders the evaluation more accurate. However, images that the model could not align were not considered, as it is impossible to store empty arrays. Nonetheless, the new code for the comparison of the faces in the images maximized the distance of the images mentioned above with a value of 4.00, as shown in Table 4. There were fewer raw images that the model was unable to align and compare than deep-funnelled images because the latter had been turned, and their resolution was lower than that of the raw images.

**Table 4.** Number of images that the model could not align per dataset.

Dataset	Total number of images	Number of images not aligned
LFW-raw	13 233	60
LFW-deep funnelled	13 233	65
SCface	4 166	1 065
ForenFace	2 819	1 197

Table 5 lists the number of images per dataset in which the model was unable to find a face during the comparison of the images. A total of 25.6% of the images of SCface and 42.5% of those of ForenFace could not be aligned by the model, and no face was identified in them [28]. In general, the face detector could not find faces when the selected landmarks of the face were absent, such as in side profiles or low-resolution images.

**Table 5.** Number of images on which the nn4.smal2.v1 model could not find a face per dataset.

Dataset	Total number of images	Number of images no face
LFW-raw	13 233	57
LFW-deep funnelled	13 233	65
SCface	4 166	1 067
ForenFace	2 819	1 197

**Figure 2.**
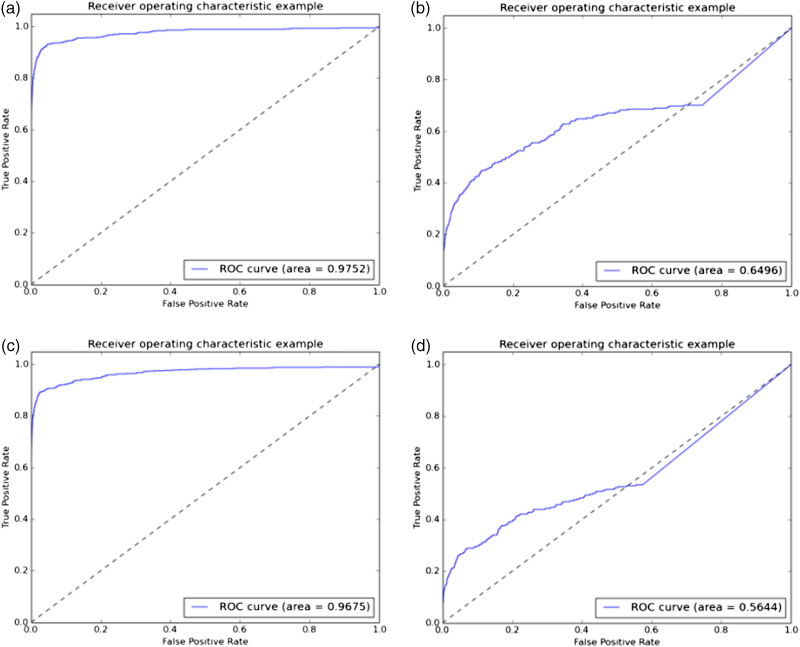
Receiver operating characteristics (ROCs) of nn4.small2.v1 OpenFace model with the use of Raw (A), Raw LFW dataset (B), SCface dataset (C) and ForeFace dataset (D)

Figure 2 as created based on the new evaluation code for the nn4.small2.v1 model. The difference in ROCs between Figure 2 (A)–(E) and the results in Table 3 obtained because two evaluation codes were used. The figures shows the ROCs per dataset while using 10 random processes for each. According to Figure 2 (C) and (D), it is clear that there was a trend of discontinuity in the curves after a certain point. This can be attributed to the maximization of the initial distances of all images in which the detector could not find a face, with a value of 4.0.

Thus, for both the ForenFace and SCface datasets, the FPR and TPR should have been almost identical once the detector had processed all images that it could. The images that the detector could not align are not considered. Moreover, the input images from the ForenFace and SCface datasets were different in size from those of the LFW datasets, which was the size required by the detector. This means that the images needed to be rescaled while the detector processed them. This could have affected the performance of both the detector and the models, and might have contributed to the discontinuity of the relevant curves.

Only 10 processes were used for the evaluation of the model, because, as Table 6 shows, it was time consuming to obtain the relevant results. Moreover, a considerable number of processes (two more than in the evaluation of Amos [4]) were needed to obtain representative performance from the model. For these processes, 10 random images of different subjects were compared from each dataset with all images of the relevant dataset, including the same image. Table 7 shows the performance of the models based on the new evaluation code using the LFW-raw dataset. This clarifies that there were slight differences between the results of evaluation approaches in terms of the AUCs of the OpenFace models. These differences can be attributed to the random sampling of the images used for the evaluations. Furthermore, according to the runtimes of the models, it is clear that model nn4.small2.v1 was the fastest. This was expected, as the nn4.small1.v1 considered the second smallest number of parameters after the nn4.small2.v1 model. However, the exponential rise in the sizes of image databases provides additional value to the expected runtimes of the models. Based on the above, an in-depth examination and report only of the ROCs of the nn4.small2.v1 model is provided here.

**Table 6.** Calculated equal error rate (EER), area under curve (AUC) and threshold per dataset of nn4.small2.v1 OpenFace model. ($\bar{x}$ ± s)

Dataset	EER	AUC	Threshold
LFW-raw	0.068 66 ± 0.068 66	0.974 56 ± 0.026 57	0.750 39 ± 0.523 50
LFW-deep funnelled	0.177 81 ± 0.228 06	0.907 36 ± 0.111 17	0.776 68 ± 0.112 39
SCface	0.359 20 ± 0.011 32	0.654 34 ± 0.015 93	0.652 29 ± 0.004 79
ForenFace	0.392 32 ± 0.036 73	0.614 24 ± 0.040 08	0.480 53 ± 0.019 78

**Table 7.** Calculated equal error rate (EER), area under curve (AUC) and the threshold of OpenFace models using the LFW-raw dataset. **(**$\bar{x}$ ± s)

Model	EER	AUC	Threshold	Runtime (min)
nn4.v1	0.201 26 ± 0.05	0.872 66 ± 0.50	0.893 60 ± 0.03	208.328 ± 4.97
nn4.v2	0.072 18 ± 0.04	0.977 71 ± 0.02	0.803 34 ± 0.03	194.844 ± 2.20
nn4.small2.v1	0.068 66 ± 0.06	0.974 56 ± 0.03	0.750 39 ± 0.05	147.694 ± 3.89
nn4.small1.v1	0.078 78 ± 0.06	0.971 43 ± 0.03	0.735 88 ± 0.05	157.426 ± 2.23

The performance of nn4.small2.v1 model on each dataset clarified the robustness of the correlation between the performance of the model and the dataset used. The resolution of the input images played an important role in the evaluation of the models. Table 6 shows the calculated EER, AUC, and the threshold to be used per dataset to obtain more accurate verification/recognition results with using the nn4.small2.v1 OpenFace model. It is evident that the EER increased as the quality/resolution of the input images decreased.

The above-mentioned results lead to the conclusion that the performance of the model is dependent on the quality/resolution of the input images. Table 8 shows the amount of time that the model needed per dataset for a one-by-one image comparison and one image comparison with the full the relevant dataset. In general, the runtime depended on the quality and number of input images. However, as the quality of the images decreased, there was a stronger correlation between the runtime of the model and the quality of the images than between it and the number of images.

**Table 8.** Runtime of nn4.small2.v1 model per dataset for one-by-one image comparison, and a comparison with the full relevant dataset.

Dataset	Number of images	Runtime ($\bar{x}$ ± s)	
		One by one (s)	One by dataset (min)
LFW-raw	13 233	2.912 14 ± 0.133 64	147.694 ± 3.89
LFW-deep funnelled	13 233	2.829 15 ± 0.103 33	140.556 ± 10.01
SCface	4 166	8.160 33 ± 5.965 04	207.976 ± 3.83
ForenFace	2 819	5.984 54 ± 4.085 99	53.506 ± 13.06

As Tables 4 and 5 show, SCface and ForenFace had a smaller number of images compared to LFW, and contained more images that the model had been unable to align, and in which it had been unable to find a face. This can be attributed to the fact that the model needed more time to process images of low resolution as the number of images that it could not align, or in which it could not find a face, increased. Based on the results in Table 6, it is clear that the threshold reported in [1] was not a good approximation for any of the tested datasets because it was higher than the calculated threshold. Moreover, even for raw and deep-funnelled LFW datasets, the calculated threshold was notably lower than the 1,1 in [1]. Therefore, the threshold should depend on the dataset used.

As the quality of the input images decreased, the proper threshold decreased as well. In Tables 7 and 8, the calculated threshold is approximately 0.35 points lower than that reported for FaceNet using LFW datasets. Moreover, the thresholds for the SCface dataset were approximately 0.65 and 0.48 for the ForenFace. Therefore, there was a reduction of approximately 50% in the initial threshold using SCface and ForenFace. Based on the results on SCface and ForenFace, it is evident that there was a noticeable FPR for both, which means that in forensic use, the result of OpenFace could have been in favour of the suspect, which is not objective. This clarifies the crucial role of an appropriate threshold for adequate results of the comparison of images, as the verification depends solely on the calculated L2-norm distance. Nevertheless, for both the SCface and ForenFace datasets, a considerable number of images were not aligned.

A comparison of the two evaluations of the models of OpenFace on the LFW datasets shows that the runtime of the models relied heavily on the machine used for the experiments. On an NVIDIA Corporation GM204 [GeForce GTX 980] GPU with Ubuntu 14.04, the runtime refers to the scale of seconds, whereas that reported in [4] referred to a scale of milliseconds (1 s = 1 000 ms).

The above implies that as the size of the dataset increased, the relative runtimes for a query image to the dataset rose proportionally. Moreover, the reported AUC of the nn4.small2.v1 model was 0.973, whereas the AUC obtained using the new evaluation code on the same dataset decreased slightly. Furthermore, the number of images that the models could not align corresponded to that reported in [4]. Nevertheless, there was no reference in [4] to the number of images in which the models could not find a face using the LFW benchmark. There is a major difference between the two evaluation approaches: The one reported in [4] considered both the LFW datasets simultaneously, whereas the proposed evaluation approach examined each dataset separately. This could have had a slight effect on the results, as when the model could not align the raw LFW image, it considered the relevant deep-funnelled LFW image. However, this does not justify the magnitude of the difference between the approaches in terms of the performance of the models.

OpenFace was further examined regarding the performance of its models based on various image modifications. In particular, the following image modifications were examined: (1) 90° rotation, (2) 180° rotation, (3) vertical flip, (4) horizontal flip, (5) cropping, (6) resizing to smaller dimensions and (7) resizing to larger dimensions. All the examined initial images were 250 × 250 pixels. The experiments show that when an image was rotated or flipped vertically, the models were unable to find a face in them. Moreover, when the alignment of the input image did not normally result in a tilted image, the models were not always able to find a face on such images either. This depended on the degree of tilt and the resolution of the image. However, the models performed relatively well when the images were flipped horizontally, on a scale of 0.3 raised to the relevant initial distance between the query images.

For modifications to sizes of the images, multiple dimensions between 100 × 100, and 600 × 600 pixels were used. The models perform sufficiently well after the above two modifications. Specifically, for resized images, either smaller or bigger, there was an increase in scale of 0.002–0.01 in the relevant initial distance. For cropped images, there was an increase in scale of 0.015 in the relevant initial distance.

Another useful part of OpenFace is the classifier.py code. This considers each input image separately and calculates the confidence (between 0.0 and 1.0) with which the dlib [18] can classify the query face in the image. The experiments show that when the confidence of the classifier for a query image was lower than 0.5, the EER was high and the AUC was small. In general, a significant number of images in all examined datasets yielded classification confidence scores of smaller than 0.5. However, the number of these images increased when the SCface and ForenFace datasets were used, as the resolution of the relevant images was lower than those in the LFW datasets. Therefore, when facial comparison between a query and a reference image was needed, the use of the classifier yielded a reasonably good estimation of the adequacy of the results of the comparison. This can be the first part of the procedure if OpenFace is used for forensic purposes. Then, based on this score, whether OpenFace per query image should be used can be determined, as the evaluation of models of OpenFace depends significantly on the input images.

## Discussion

There are multiple advantages of using OpenFace compared with other facial analysis toolkits. As described in [29] and [2], a trained model is available online, no specific hardware is needed, and it allows the user to replace or alter its methods freely. For instance, the OpenFace face point detector can be replaced to further improve the performance of OpenFace on images with low resolution as well as side profile images. However, the use of other landmark points can be further examined, which can help detect landmarks in side profile images. Furthermore, the performance of the detector on tilted images can be improved with a few modifications, such as better alignment of the image. Moreover, all the available models, for both comparison and classification, can be retrained. A drawback of the retraining of models is the need for a large dataset of 500 000 images in jpg or png format in folders per subject.

However, no official report has examined the existence of duplicates in the training dataset, which should be further studied as they can improve the performance of OpenFace models. Further, regarding computational and memory-related demands in retraining the classification model, CUDA needs to be used. Moreover, the retraining of the models for forensic purposes can be based on the relevant forensic data, which has not yet been researched. There is no open-source dataset of this type that is as large as is needed at present.

An important result of this study is the noticeable correlation between the resolution of the input images and the performance of OpenFace. However, the degree of this correlation should be examined further. The aforesaid leads us to infer that OpenFace is currently inadequate for use for forensic purposes, as the resolution of the input images is intimately related to its performance.

Nevertheless, research on the use of the likelihood ratio (LR) concerning the calculated distances for query faces can help improve the results of OpenFace because the similarity among the queried faces can be determined based on a statistical scale, as with the LR used for DNA profile comparison. To determine the proper LR scale, various factors can be considered, such as the quality and the size of both query images, the racial and sex group to which people in the images belong, and the interval between the query images. In general, with the use of the LR, the use of OpenFace may prove more feasible in forensics. Often, the LR in forensic face examination is defined as the hypothesis (of the prosecutor) that the face in the given image is identical to that of the suspect, against the hypothesis (of the defence) that the faces are not the same.

Another research question that needs further examination is the best choice of model based on the query data and its application based on the performance of the models. Moreover, the runtime of OpenFace models was significantly longer than that reported for Amos, which could be attributed to different hardware. However, an examination of runtime should be undertaken to revalidate the results on a different machine, ideally a GPU. Furthermore, OpenFace has only been officially tested on Linux and OSX machines. However, the proper installation of all OpenFace requisites is possible in principle on Windows as well [29].

A remarkable aspect of OpenFace models is that there was a noticeable performance degradation on input images of Asian people and children. This can be attributed to the training datasets, which were probably imbalanced in terms of racial and age groups. However, this is an issue that can be simply eliminated by training the models on balanced datasets. Another interesting research topic for future research is the examination of OpenFace using images of people across a large age interval. There is a need for investigations on facial recognition based on skin and age variations, which are useful in cases involving missing children, for instance, whose appearance changes rapidly over a few years.

## Conclusion

In this article, OpenFace was examined in relation to face verification, recognition and clustering tasks on still images based on multiple datasets. Its performance was verified on the LFW benchmark datasets. However, in light of use in forensics, OpenFace minimized the threshold reported in [1] and, its performance depended on the quality of the dataset used. Moreover, the runtime of the models depended on multiple factors: namely, the number of model parameters, the quality of the machine, and the size and quality of the dataset. A retraining of the models can improve the performance of OpenFace on the relevant forensic datasets. Using training datasets balanced in terms of age and racial diversity is an example of such retraining.

The effect of the quality of the query images on the efficiency of OpenFace was apparent. Therefore, OpenFace is inadequate in its current state for use in forensics applications owing to the low quality of images acquired from closed-circuit television (CCTV)—a major source of images used in the area. For better quality images, the system works well and can be considered a complementary tool in forensic examination.

## References

[1] Schroff F, Kalenichenko D, Philbin J FaceNet: A unified embedding for face recognition and clustering. IEEE Conf Comput Vis Pattern Recognit; 2015 Jun 8–10; Boston (MA). 2015 p. 815–823.

[2] Amos B, Harkes J, Wang J, et al. OpenFace models. GitHub [Internet]; 2016 [cited 2016 Jan 27]. Available from: https://github.com/cmusatyalab/openface/tree/master/models/openface

[3] Heath K, Guibas L FaceNet: Tracking people and acquiring canonical face images in a wireless camera sensor network In: 1st ACM/IEEE international conference on distributed smart cameras. IEEE; 2017 Sept 25–28; Vienna, Austria. 2007 p. 117–124.

[4] Amos B, Harkes J, Wang J, et al. Demo 1: Real-time web demo. GitHub [Internet]; 2016 [cited 2016 Jan 27]. Available from: http://cmusatyalab.github.io/openface/demo-1-web/

[5] Weinberger KQ, Blitzer J, Saul LK Distance metric learning for large margin nearest neighbor classification. J Mach Learn Res. 2009;10:207–244.

[6] Schultz M, Joachims T Learning a distance metric from relative comparisons. In: Neural Information Processing Systems (NIPS) 16. Cambridge: MIT Press; 2004 p. 41–48.

[7] Rumelhart DE, Hinton GE, Williams RJ Learning representations by back-propagating errors. Nature. 1986;2:533–536.

[8] LeCun Y, Boser B, Denker JS, et al. Backpropagation applied to handwritten zip code recognition. Neural Comput. 1989;1:541–551.

[9] Duchi J, Hazan E, Singer Y Adaptive subgradient methods for online learning and stochastic optimization. J Mach Learn Res. 2011;12:2121–2159.

[10] Lin M, Chen Q, Yan S Network in network. arXiv Preprint. 2013;3:10.

[11] Szegedy C, Liu W, Jia Y, et al. Going deeper with convolutions. CoRR, abs/1409.4842; 2015 Jun 7‐12; Boston (MA). 2014:1–12.

[12] Zeinstra CG, Veldhuis RNJ, Spreeuwers LJ, et al. ForenFace: a unique annotated forensic facial image dataset and toolset. In: IET Biometrics. 2017;6:487–494.

[13] Grgic M, Delac K, Grgic S SCface – Surveillance cameras face database. Multimed Tools Appl. 2011;51:863–879.

[14] Bradski G OpenCV. Dr. Dobb’s journal of software tools [Internet]; 2000 [cited 2016 Jan 27]. Available from: http://opencv.org/

[15] Collobert R, Kavukcuoglu K, Farabet C Getting started with torch; 2011 [cited 2016 Jan 27]. In: BMVC 2015. Available from: http://torch.ch/docs/getting-started.html#_

[16] Preston-Werner T, Wanstrath C, Hyett P How people build software. Docker [Internet]; 2008 [cited 2016 Jan 27]. Available from: https://github.com/

[17] Hykes S Docker [Internet]; 2013 [cited 2016 Jan 27]. Available from: https://www.docker.com/

[18] Chen D, Ren S, Wei Y, et al. Joint cascade face detection and alignment. Springer International Publishing 2014;8694:109–122.

[19] Huang GB, Ramesh M, Berg T, et al. Labeled faces in the wild: A database for studying face recognition in unconstrained environments. Univ Massachusetts Amherst Tech Rep. 2007;1:7–49.

[20] Baltrušaitis T, Robinson P, Morency LP OpenFace: an open source facial behavior analysis toolkit. IEEE Winter Conf Appl Comput Vis; Mar 7–10; Lake Placid (NY). 2016:1–10.

[21] King DE Dlib-ml: A machine learning toolkit. J Mach Learn Res. 2009;10:1755–1758.

[22] Vision & Interaction G. FaceScrub. Vision & interaction groupe; 2014 [cited 2016 Feb 27]. Available from: http://vintage.winklerbros.net/facescrub.html

[23] Yi D, Lei Z, Liao S, Li SZ Learning Face Representation from Scratch. arXiv: 1411.7923, 2014.

[24] Zeiler MD, Fergus R Visualizing and understanding convolutional networks. Comput Vision – ECCV. 2014;8689:818–833.

[25] Hanley A, Mcneil J, Ph D The meaning and use of the area under a ROC curve. Radiology. 1982;143:29–36. 7063747 10.1148/radiology.143.1.7063747

[26] Mayoue A Biosecure tool eyes position detector; 2007 Available from http://schema.org/SearchResultsPage

[27] Sarlis NV, Christopoulos SRG Visualization of the significance of receiver operating characteristics based on confidence ellipses. Comput Phys Commun. 2014;185:1172–1176. doi: 10.1016/j.cpc.2013.12.009

[28] Amos B, Harkes J, Wang J, et al. OpenFace. GitHub [Internet]; 2016 [cited 2016 Jan 27]. Available from: https://cmusatyalab.github.io/openface/

[29] Cournapeau D Scikit-learn [Internet]; 2007 [cited 2016 Jan 27]. Available from: http://scikit-learn.org/stable/

